# Toward Equitable Health Care: Bridging the Gap in the Health of Incarcerated Individuals in Africa

**DOI:** 10.1002/puh2.70020

**Published:** 2024-12-21

**Authors:** Praise Oyedepo Okunlola, Abdulhammed Opeyemi Babatunde, David Mobolaji Akoki, Opeyemi Temitope Ilori, Victor Oluwafemi Femi‐Lawal, Favour Mofiyinfoluwa Abiona, Samuel Tobi Tundealao

**Affiliations:** ^1^ Faculty of Dentistry College of Medicine University of Ibadan Ibadan Oyo State Nigeria; ^2^ Faculty of Clinical Sciences College of Medicine University of Ibadan Ibadan Oyo State Nigeria; ^3^ Department of Biostatistics and Epidemiology Rutgers School of Public Health Piscataway New Jersey USA; ^4^ Cancer Health Equity Center for Excellence Rutgers Cancer Institute New Brunswick New Jersey USA

**Keywords:** Africa, developing country, equitable health coverage, healthcare, incarcerated

## Abstract

The situation of correctional facilities in African countries represents a critical threat to health due to overcrowding, poor living conditions, and limited access to medical services. With over 3000 facilities and nearly a million incarcerated individuals, the prevalence of health conditions such as HIV/AIDS, mental health disorders, and tuberculosis is alarmingly high. These conditions are exacerbated by physical and psychological abuse and inadequate healthcare infrastructure. Despite these challenges, the health needs of incarcerated individuals in Africa remain largely neglected. This article provides a review of the health status of incarcerated individuals in Africa, drawing on limited available data. Lessons from developed countries highlight the potential for effective interventions through structured healthcare programs and policies. Recommendations include adopting the World Health Organization (WHO) prison health framework, improving judicial efficiency to reduce overcrowding, ensuring healthcare is managed by health ministries, and establishing rehabilitation centers. These measures are crucial for integrating incarcerated individuals back into society and achieving equitable health coverage in Africa.

## 1 Introduction

The harsh reality of the system of correctional facilities in African countries is a threat to health. There are about 3000 correctional facilities and a million incarcerated individuals across Africa [1]. Despite this failing system, incarceration rates continue to increase, whereas crime rates continue to decrease in the continent [2]. In countries like Uganda and Zambia, correctional facilities hold three times their maximum capacity [3].

The failing correctional system contributes to a high prevalence of health conditions of public health concerns such as HIV/AIDs, mental health disorders, tuberculosis, and others among incarcerated individuals. For instance, the prevalence of mental illness is five times higher among incarcerated individuals in Nigeria and South Africa than the general population [4, 5]. Likewise, the prevalence of HIV infection among incarcerated individuals ranged from 2.3% to 34.9% in a study carried out across 24 Sub‐Saharan African countries [6]. Low socio‐economic status, poor living conditions, physical assault, and psychological abuse inside correctional facilities make incarcerated individuals more vulnerable to these conditions [7]. Besides, there is a huge treatment gap for these conditions and limited health prevention and promotion interventions for incarcerated individuals, and the well‐being of incarcerated individuals has received limited attention in several African countries and the global health community.

Achieving equitable health coverage requires unbiased and affordable access to quality healthcare for everyone, including vulnerable populations. In many African countries, however, the necessary infrastructure and resources to support equitable health coverage remain underdeveloped, making it challenging to meet the goal of equitable health coverage by 2030 [8]. Within this context, incarcerated individuals face even greater disparities, as they are often excluded from or have limited access to essential healthcare services [8]. This disproportionate impact underscores the urgent need for targeted interventions and policy reforms to ensure that health systems are inclusive and prioritize the needs of all individuals, regardless of their circumstances. This perspective article aims to provide a review of the health of incarcerated individuals in Africa and offer recommendations for achieving equitable health coverage, drawing on lessons learned from more developed countries. Due to Africa's vastness and diverse geopolitical and religious principles, it is ineffective to group the correctional systems of all African countries together. However, this perspective article represents an initial step in the evaluation of equitable healthcare in correctional systems in Africa.

## 2 Review of the Current Situation

There is a paucity of data and research publications on the state of correctional facilities in Africa [9]. The limited research and resources dedicated to the health of incarcerated individuals paint an incomplete picture of the status of the healthcare of correctional facilities. These correctional facilities, often remnants of a colonial past, are plagued by overcrowding, malnutrition, and a disregard for basic human rights [1]. This creates a breeding ground for infectious diseases, jeopardizing the health of incarcerated individuals, staff, and, ultimately, the communities they return to. Factors such as the high number of individuals awaiting trial and a measure of judicial efficiency contribute to Nigeria's poor state of custodial centers [9]. Although the picture is similar across Africa, countries like Comoros and South Africa are at the extremes [3]. As far as we know, Comoros has 225 incarcerated individuals in three correctional facilities, and at the other end of the spectrum, South Africa has about 235 facilities holding close to 160,000 incarcerated individuals at an average size of 657 incarcerated individuals per facility [1, 3].

The evaluation of prison conditions in North Africa is significantly obstructed by most of the government's refusal to allow independent monitoring or disclosure of fundamental information on prisons [10]. A few countries in the region permit human rights organizations access to their correctional institutions [10]. Despite ongoing efforts throughout North Africa to align criminal justice systems with international standards and human rights law, substantial hurdles to reform persist [11]. These factors encompass prison congestion, delays in the trial process, inadequate coordination among criminal justice agencies, insufficient resources in some countries of political will, and a lack of credible statistics regarding prison systems [11].

The correctional facilities in Sub‐Saharan Africa are largely male‐dominated, with the female population, although increasing over the years, still forming a minority [12]. These facilities often concentrate huge numbers of incarcerated individuals with HIV/AIDS and others who are susceptible to the infection [13]. These individuals have poor access to basic healthcare and increased tendencies for risky behaviors. These factors, coupled with the poorly maintained infrastructure, enable the spread of diseases and epidemics in correctional centers. For instance, it is estimated that correctional facilities in Africa bear between 6 and 30 times the prevalence of tuberculosis than the general population [14].

Previous studies have identified factors such as unhygienic overcrowding, unhygienic conditions, and poor sanitation, with most penal institutions failing to meet the minimum standards [9, 15]. One of the studies also found that about 17 countries in Africa incarcerated young people in the same facilities as adults, exposing them to higher risks of physical violence and sexual abuse [9]. Another study conducted in Zambian correctional centers reported prevalent vulnerability among young, incarcerated persons due to lack of financial support, leaving them vulnerable to manipulation by wealthier and more powerful older incarcerated individuals who may sexually abuse them [16]. Other issues affecting incarcerated individuals, as noted in the review, included poor food quantity and quality and a lack of access to healthcare and basic necessities [16].

Despite the poor access to healthcare, some incarcerated individuals received the first health education on HIV and other diseases of public health concern in the correctional facility [17]. As an institution of government, some correctional facility systems still function in promoting health and well‐being, especially through collaboration with nongovernmental organizations (NGOs) and healthcare bodies [17]. Similarly, the need for encouragement from correctional officers and other incarcerated individuals to seek HIV testing and treatment speaks to potential barriers to accessing these services [13]. The structured nature of life in a correctional center, helpful for planning clinic visits and medication refills for HIV/AIDS and tuberculosis, might not translate to other health needs [13]. Government resources are stretched thin, and alternative sources of health services are NGOs, which also have to collaborate with the already overwhelmed government health structures for access to this population [13].

## 3 Lessons From Non‐African Countries

Several programs and interventions have been implemented in high and middle‐income countries to improve the health outcomes of incarcerated individuals. The designs and target populations of these interventions vary, ranging from programs within the correctional centers to interventions for formerly incarcerated people who are re‐entering the community settings [18]. In the United Kingdom and some other European countries, the treatment of incarcerated individuals is often under the Ministry of Justice or jointly with the Ministry of Health [19]. However, comprehensive programs such as HIV screening tests and comprehensive health information are available to all incarcerated individuals. In several countries, HIV/AIDS patients are also entitled to psychological support [19]. Sexually transmitted disease screening levels are often much lower, however. These tests are compulsory for incarcerated individuals in a few countries, such as France, Lithuania, Poland, and Hungary [19]. In most countries, psychologists are available to provide treatment for incarcerated individuals. A few countries, such as Portugal and France, also have special psychiatric institutions for mentally challenged incarcerated individuals [19].

The Nordic countries, known for their robust universal healthcare systems and focus on social equity, offer valuable lessons in healthcare provision for incarcerated individuals [20]. In these countries, incarcerated healthcare is generally included in the national healthcare system instead of being administered by correctional services, thereby providing parity of care between incarcerated individuals and the general populace [20]. In Norway, municipal health services within correctional institutions administer healthcare services, ensuring access to general practitioners, mental health workers, and specialists when required. This method mitigates stigmatization and guarantees continuity of care post‐release [21, 22]. Mental health interventions are a significant focus, with programs emphasizing rehabilitation over punishment. In Sweden and Finland, specialist psychiatric units serve incarcerated individuals with serious mental diseases, emphasizing therapeutic treatment over confinement [23]. Furthermore, harm reduction measures, including needle exchange programs and opioid replacement therapy, are employed to tackle substance use disorders in prisons, therefore substantially decreasing the risk of infectious disease transmission and improving reintegration outcomes [23]. These practices demonstrate the potential of integrated and equitable healthcare systems to improve the health and rehabilitation of incarcerated populations.

In the United States, diverse interventions have been implemented to improve mental health outcomes, with results ranging from no change to significant improvement in treatment targets. A system training for emotional predictability resulted in improvements in emotion and behavior management, and a clinical alternative to punitive segregation intervention reduced self‐harm levels [24]. Several other interventions produced mixed results, such as a medication course at a youth correctional facility or a correctional intervention to help women recover from trauma [25]. To address overcrowding, many US states have implemented policies emphasizing alternatives to prison, including diversion programs, parole reforms, and early release initiatives for nonviolent offenders [26]. These initiatives seek to decrease the prison population while prioritizing rehabilitation over retribution [26]. Correctional facilities have instituted screening, vaccination, and treatment programs to combat communicable health conditions. Initiatives such as the Affordable Care Act have enhanced the continuity of treatment for incarcerated individuals transitioning back into their communities [27]. The emphasis on juveniles has transitioned to rehabilitative methods, as some US states have implemented specialized juvenile justice systems that prioritize education, mental health assistance, and community‐based alternatives to detention [28]. Programs like trauma‐informed care and skill‐building seminars seek to diminish recidivism and enhance outcomes for juvenile offenders [28]. In Eastern Europe and Central Asia, management of HIV and other sexually transmitted diseases includes services such as early detection, counseling, testing, and treatment. Continuity care after release is also a key part of these interventions [29].

Although lessons from other countries provide valuable insights, several factors limit their applicability to African contexts. Resource constraints, overcrowded correctional facilities, and underfunded systems hinder the implementation of comprehensive programs. Cultural differences, such as stigma around mental and sexual health, reduce the effectiveness of interventions like counseling or disease screening. Unlike regions where health ministries manage prison healthcare, many African nations rely on justice departments, leading to inefficiencies. Additionally, the lack of robust data on incarcerated populations and the unique challenges of stigma and reintegration complicate the adoption of external models. Judicial delays further exacerbate overcrowding and health crises, underscoring the need for tailored, context‐specific solutions.

## 4 Recommendations

In pursuing equitable healthcare objectives described by the World Health Organization (WHO), addressing health inequities affecting key disadvantaged populations, such as incarcerated persons, is important. Targeted interventions for the health and welfare of incarcerated individuals are essential because of their conditions, especially regarding shared jurisdiction. This means typical interventions implemented in other vulnerable and general populations may not apply to incarcerated individuals. Therefore, it is important to prioritize these populations through context‐specific interventions. There is a need for the adoption of the WHO prison health framework and the development of a national framework by each African country [30]. This will help reduce the inequalities and improve the quality of healthcare delivery in correctional centers. In addition, the implementation of the standard minimum guidelines for the treatment of incarcerated individuals, known as “the Nelson Mandela Rules,” could facilitate equitable health among incarcerated individuals [31]. This document from the United Nations Office on Drugs and Crime outlines a range of rules concerning health as baseline treatment regarding incarcerated persons while serving their time [31].

As incarcerated individuals are released, it is also important to provide adequate mechanisms to integrate them properly with society. Comprehensive interventions, including not only healthcare services but also mental health support, substance abuse treatment, and access to education and job training, are needed to address these concerns, in addition to other burdens of stigma due to race, economic background, and substance use issues [32, 33]. There is also evidence to suggest that preventive measures for sexual health among incarcerated persons can lead to cost savings. A study at the Los Angeles County men's correctional facility showed that a screening, treatment, and condom provision intervention could significantly avert chlamydia, gonorrhea, and syphilis infections, reducing costs that would have been incurred otherwise [34].

A study in Myanmar also found that cultural perceptions such as perceptions of masculinity may significantly affect willingness to receive mental health therapy, even when available [35]. In creating programs to address these challenges, it is vital to address these cultural perceptions. Data on mental health interventions for many African countries are limited. Hence, there is need for more community‐based interventions and pilot studies to effectively develop mental health interventions for incarcerated individuals and returning citizens.

Moreover, there is a need for strict adherence to the standard capacity of correctional centers. This will ensure that they are not overcrowded. This can be achieved by facilitating the judiciary processes to reduce the waiting time for final judgment and appeals for offenders. Besides, the age bracket should be considered in allocating incarcerated individuals to rooms. Adolescent offenders should be sent to rehabilitation or remanded homes rather than correctional centers.

The health care provision in the correctional centers should be entirely supervised and provided by the Ministry of Health rather than the Ministry of Justice. This will provide a concerted effort and strengthen the provision of healthcare for incarcerated individuals. Besides, there is a need for a dedicated pool of funds for financing essential care in correctional centers, especially sexual and reproductive health and mental health. This could be from public donations, health insurance, fine and litigation fees, and donations from religious and NGOs. There is a need for the establishment of rehabilitation centers by the government for incarcerated individuals after serving their jail term or the provision of policy enforcing private rehabilitation centers to create special packages for persons who have just served a jail term. This includes medical, social, and vocational rehabilitation to ease the community integration process. Finally, there is a need for an effective disease surveillance system and routine evaluation of the health care services in correctional facilities to maintain the standards.

Another recommendation is to ensure thorough exploration and advocacy for the minimum age of criminal responsibility, as research indicates that children as young as 10 should not be incarcerated due to their developmental stage and mental maturity, as well as the detrimental effects on their health and well‐being, both presently and in the future. Detailed information on the recommended strategies on how to ensure equitable health care among incarcerated individuals in Africa is presented in Table 1.

**TABLE 1:** Barriers and strategies to improve healthcare in African correctional facilities.

Barriers to improved healthcare	Strategies to improve healthcare
Overcrowding	Implement judicial reforms to reduce delays in trials and appeals
	Prioritize non‐custodial sentences for minor offenses (e.g., diversion programs, early release)
	Establish community‐based rehabilitation programs to reduce prison population
Limited healthcare infrastructure	Increase funding for healthcare infrastructure within correctional facilities
	Collaborate with NGOs and community healthcare providers to supplement prison health services
Inadequate healthcare personnel and training	Provide specialized training for prison healthcare workers on managing chronic diseases, infectious diseases, and mental health
	Recruit more healthcare professionals into correctional settings through incentives
Poor sanitation and hygiene conditions	Invest in prison facility upgrades to improve sanitation (e.g., clean water, proper waste disposal)
	Regular hygiene education programs for both inmates and staff
Stigma and cultural barriers	Promote awareness campaigns to reduce stigma related to mental health and HIV/AIDS
	Integrate mental health services into routine healthcare, making them part of general health screenings
Limited access to specialized care	Facilitate partnerships with external healthcare providers for specialized care (e.g., psychiatric services, surgery)
	Use telemedicine to connect incarcerated individuals with specialists in remote locations
Lack of health data and surveillance systems	Establish and maintain health surveillance systems to track health trends and outcomes in correctional facilities
	Collect and analyze health data to identify the most pressing health needs and allocate resources effectively
Poor nutritional and physical conditions	Improve food quality and quantity in prisons, focusing on balanced nutrition
	Develop physical rehabilitation programs that support both physical health and mental well‐being
Inadequate reintegration programs	Create rehabilitation programs that address physical, mental, and vocational skills, aiding reintegration post‐release
	Develop transitional healthcare services to ensure continuity of care from prison to community

## 5 Conclusion

Addressing the health disparities faced by incarcerated individuals in African correctional facilities is essential for achieving equitable health coverage. The severe overcrowding, inadequate healthcare, and poor living conditions create a breeding ground for many infectious diseases and mental health issues. Adopting the WHO prison health framework, enhancing judicial efficiency, and ensuring health ministries oversee correctional centers’ healthcare are critical steps. Establishing dedicated rehabilitation centers will facilitate the reintegration of formerly incarcerated individuals. Implementing these recommendations will help reduce health inequities, promote public health, and help move closer to equitable health coverage in Africa.

## Author Contributions

**Praise Oyedepo Okunlola:** conceptualization, review, and formal analysis of literature, writing–review and editing, supervision. **Abdulhammed Opeyemi Babatunde:** conceptualization, review and formal analysis of literature, writing–review and editing. **David Mobolaji Akoki:** review and formal analysis of literature, writing–review and editing. **Opeyemi Temitope Ilori:** review and formal analysis of literature, writing–review and editing. **Victor Oluwafemi Femi‐Lawal:** review and formal analysis of literature, writing–review and editing. **Favour Mofiyinfoluwa Abiona:** review and formal analysis of literature, writing–review and editing. **Samuel Tobi Tundealao:** review and formal analysis of literature, writing–review and editing, supervision.

## Ethics Statement

An ethics statement is not applicable because this article is based exclusively on published literature.

## Conflicts of Interest

The authors declare no conflicts of interest.

## Data Availability

No data are associated with this article.
